# Supplementary material for: High-depth spatial transcriptome analysis by photo-isolation chemistry
Source: Nat Commun. 2021 Jul 20;12:4416. doi: 10.1038/s41467-021-24691-8 (PMC8292322; doi:10.1038/s41467-021-24691-8)
Supplement: Supplementary file 1 — Supplementary Information [file 41467_2021_24691_MOESM1_ESM.pdf]

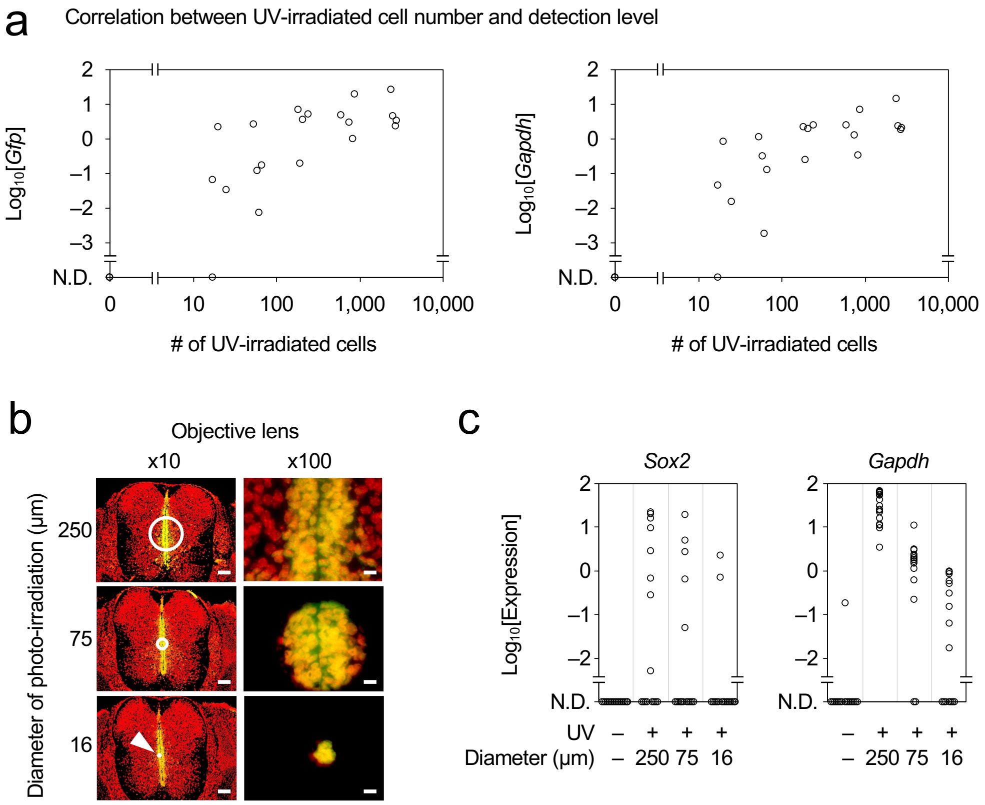

Supplementary Fig. 1 | Sensitivity of PIC with qPCR. a, Various numbers of GFP-expressing NIH3T3 cells were photo-irradiated and evaluated for the levels of *Gfp* and *Gapdh* cDNAs. **b**,**c**, Areas of various sizes in the E14.5 neural tube (**b**) were photo-irradiated and evaluated for the levels of *Sox2* and *Gapdh* cDNAs (**c**). Scale bars in **b**, 100 μm (left) and 10 μm (right).

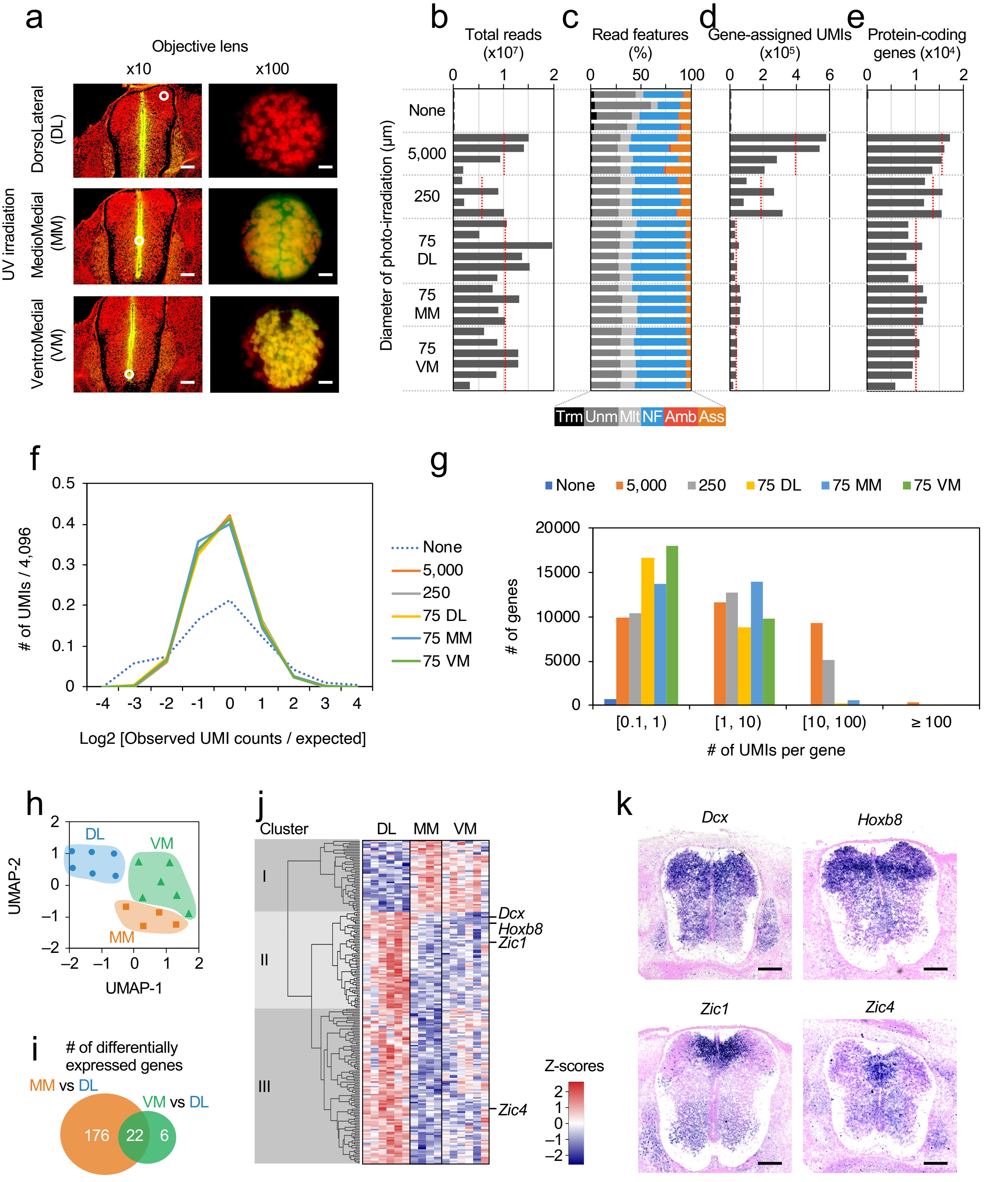

Supplementary Fig. 2 | PIC RNA-seq for neural tubes. a, Dorsolateral (DL) , mediomedial (MM) or ventromedial (VM) neural tube areas 75 µm in diameter were photo-irradiated and profiled for gene expression with RNA-seq. A representative image was shown out of the replicates (n = 4–6). **b**–**e**, Bar charts showing total read numbers (**b**), read features (**c**), counts of gene-assigned unique molecular identifiers (**d**) and detected protein-coding genes (**e**), with the red dotted lines indicating average values. Abbreviations in **c**: trimmed (Trm), unmapped (Unm), multi-mapped (Mlt), no features (NF), ambiguity (Amb) and gene-assigned (Ass). **f**, The largest portion of UMI sequences out of 4^6 (= 4,096) variations was evenly detected as expected in random distributions. **g**, Histogram showing the per-gene UMI counts. **h**–**j**, Expression profiles from DL, MM and VM domains were analysed by dimension reduction (UMAP) (**h**), and the numbers and clusters of DEGs are shown in a Venn diagram (**i**) and heat maps (**j**), respectively. **k**, Expression patterns of DEGs detected to be stronger in the DL domain by PIC were confirmed by ISH (purple, transcripts; magenta, nuclei). A representative image was shown out of three replicates. Scale bars in **a**, 100 µm (left) and 10 µm (right); **i**, 200 µm.

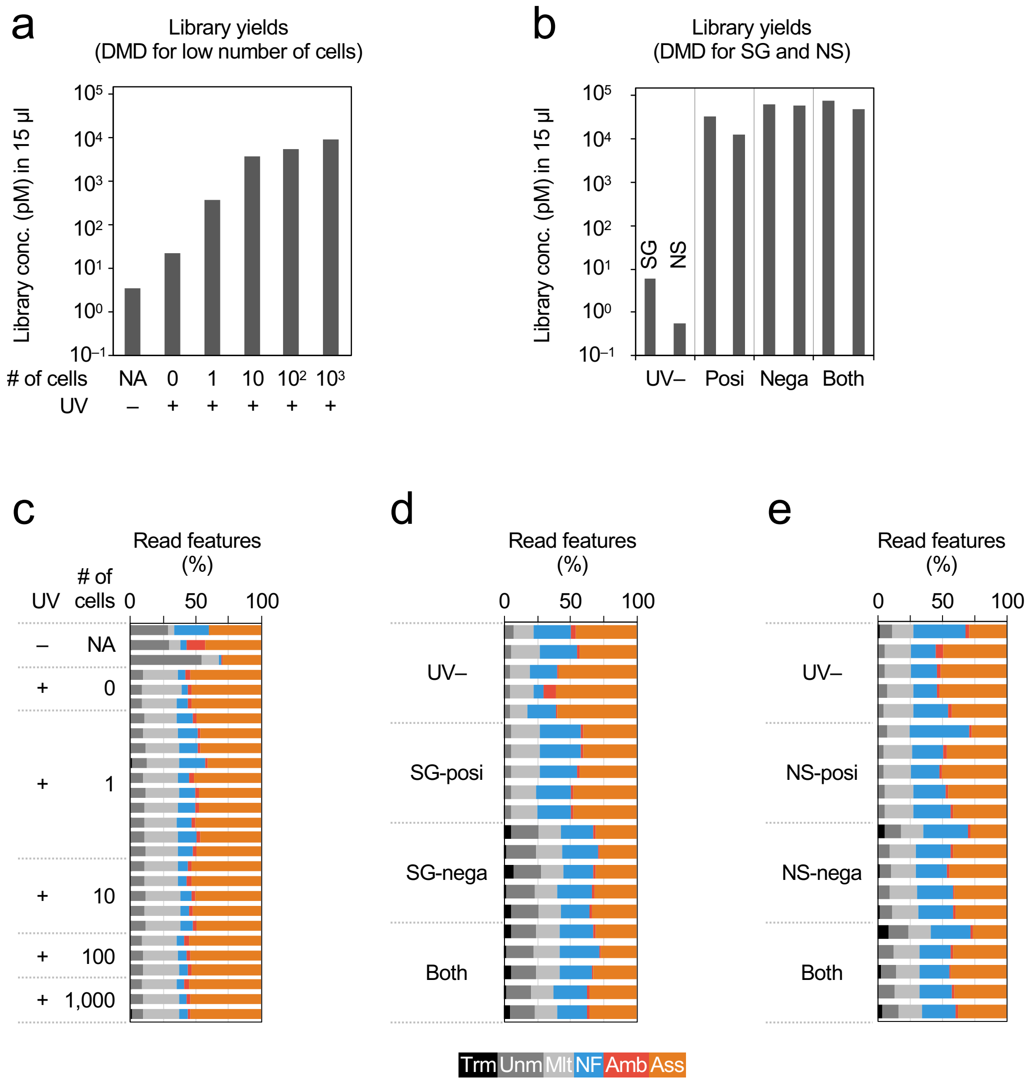

Supplementary Fig. 3 | Library yields and read features by DMD-assisted irradiations. a,**b**, qPCR analysis to measure the concentration of sequencing libraries in a total volume of 15 µl prior to sequencing, derived from DMD-assisted irradiations of a low number of cells (**a**) and of SGs and NSs (**b**). **c**–**e**, Read features of DMD-assisted irradiations of a low number of cells (**c**), SGs (**d**) and NSs (**e**).

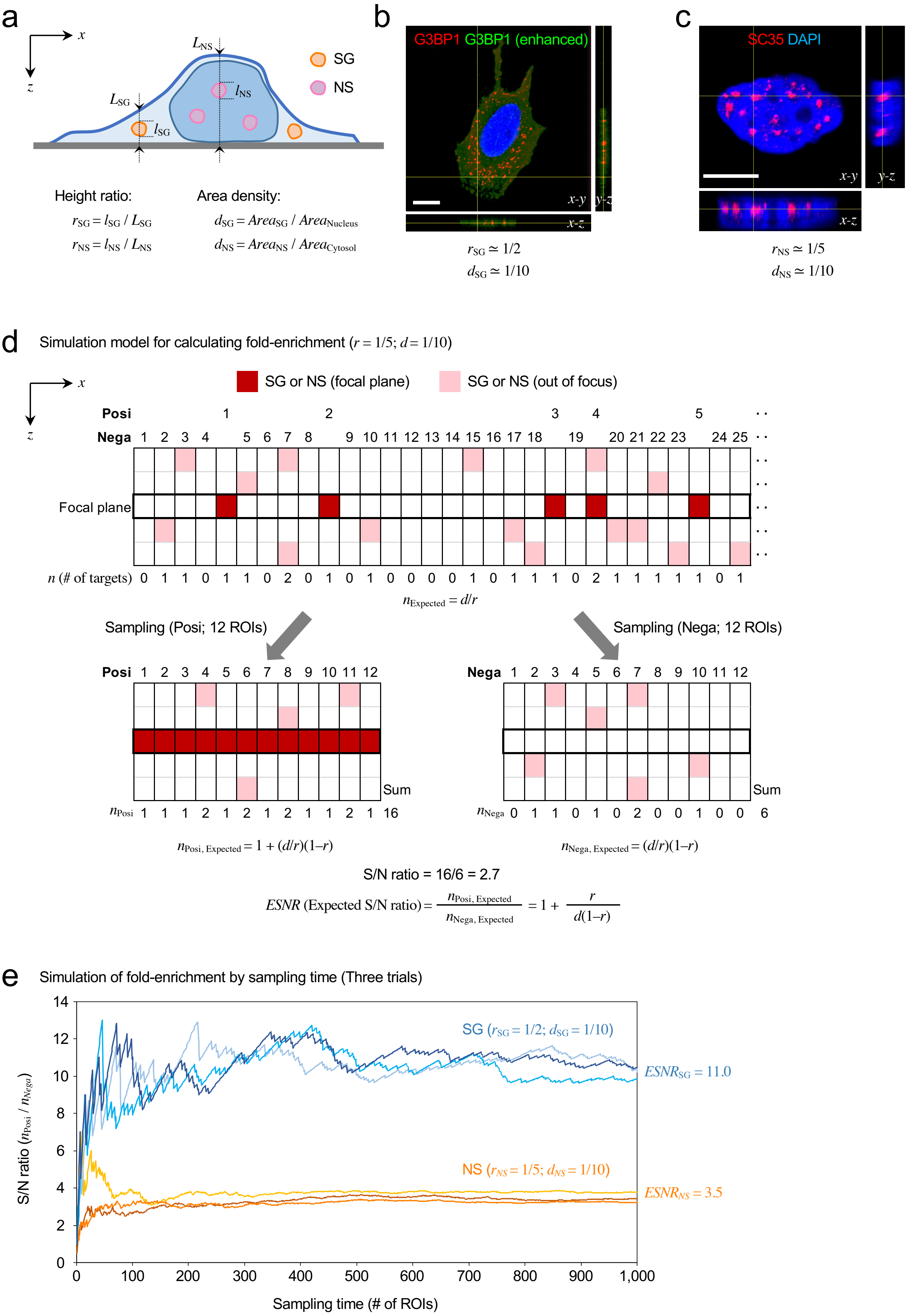
a
x
z
SG
NS
L_NS
l_NS
L_SG
l_SG
Height ratio:
r_SG = l_SG / L_SG
r_NS = l_NS / L_NS
Area density:
d_SG = Area_SG / Area_Nucleus
d_NS = Area_NS / Area_Cytosol
b
G3BP1 G3BP1 (enhanced)
x-y
y-z
x-z
r_SG ≃ 1/2
d_SG ≃ 1/10
c
SC35 DAPI
x-y
y-z
x-z
r_NS ≃ 1/5
d_NS ≃ 1/10
d
Simulation model for calculating fold-enrichment (r = 1/5; d = 1/10)
SG or NS (focal plane)
SG or NS (out of focus)
Posi 1 2 3 4 5
Nega 1 2 3 4 5 6 7 8 9 10 11 12 13 14 15 16 17 18 19 20 21 22 23 24 25
Focal plane
n (# of targets) 0 1 1 0 1 1 0 2 0 1 0 1 0 0 0 0 1 0 1 1 1 0 2 1 1 1 1 1 0 1
n_Expected = d/r
Sampling (Posi; 12 ROIs)
Sampling (Nega; 12 ROIs)
Posi 1 2 3 4 5 6 7 8 9 10 11 12
Sum
n_Posi 1 1 1 2 1 2 1 2 1 1 2 1 16
Nega 1 2 3 4 5 6 7 8 9 10 11 12
Sum
n_Nega 0 1 1 0 1 0 2 0 0 1 0 0 6
n_Posi, Expected = 1 + (d/r)(1–r)
n_Nega, Expected = (d/r)(1–r)
S/N ratio = 16/6 = 2.7
ESNR (Expected S/N ratio) = n_Posi, Expected / n_Nega, Expected = 1 + r / d(1–r)
e
Simulation of fold-enrichment by sampling time (Three trials)
S/N ratio (n_Posi / n_Nega)
0 2 4 6 8 10 12 14
0 100 200 300 400 500 600 700 800 900 1,000
Sampling time (# of ROIs)
SG (r_SG = 1/2; d_SG = 1/10)
ESNR_SG = 11.0
NS (r_NS = 1/5; d_NS = 1/10)
ESNR_NS = 3.5

Supplementary Fig. 4 | Estimation of sufficient number of ROIs. a–**c**, ROI of SG-posi or NS-posi definitely contains one signal (SGs or NSs, respectively) and may have additional signals (SGs or NSs located out of the focal plane). In addition, an ROI of SG-nega or NS-nega may contain some noise (SGs or NSs located out of the focal plane). Thus, if a small number of NS-nega ROIs are sampled, these samples may happen to be contaminated by a substantial amount of noise. We thus performed a simulation to estimate the number of ROIs to obtain stable S/N ratios. We introduced the following two parameters (**a**): height ratio (r), which is the proportion of the thickness of SGs or NSs relative to that of cytoplasm or nucleus, respectively; and area density (d), which is the proportion of the area of SGs or NSs relative to that of cytoplasm or nucleus, respectively. They were roughly estimated by confocal microscopy as shown in (**b**,**c**, a representative image was shown out of five replicates.). Scale bars,10 μm. **d**, If it is hypothesised that r is 1/5 and d is 1/10, 5 (1/r) voxels along the z-axis would be irradiated by UV per single ROI. As SGs or NSs will appear at a probability of 1/10 (d), the expected per-ROI number is thus 5/10 (d/r). In the SG-nega and NS-nega ROIs, SGs and NSs are omitted from the focal plane; thus, the expected per-ROI number ($n_{\text{Nega, Expected}}$) is 5/10 × 4/5, or $(d/r)(1–r)$. In contrast, SG-posi and NS-posi ROIs definitely include one more additional SG or NS, respectively, so the expected per-ROI number ($n_{\text{Posi, Expected}}$) is $1 + (d/r)(1–r)$. Therefore, the expected S/N ratio ($ESNR$, $n_{\text{Nega, Expected}}$ / $n_{\text{Posi, Expected}}$) is given as $1 + r/[d(1–r)]$. However, as SGs and NSs are randomly distributed in these voxels, the S/N ratio may be affected by chance. **e**, We performed a Monte Carlo simulation, in which SGs or NSs were randomly distributed in the voxels according to the measured r and d values. ROIs for posi and nega were sequentially sampled and the S/N ratios were calculated (in triplicate trials). The results showed that the S/N ratio was largely affected by chance before 100 samplings. In contrast, the S/N ratios were stable and gradually converged to $ESNR$ after 200 samplings. Given that several SGs and NSs are encompassed in a single cell, UV irradiation of several dozen cells is estimated to be sufficient to obtain a stable S/N ratio.

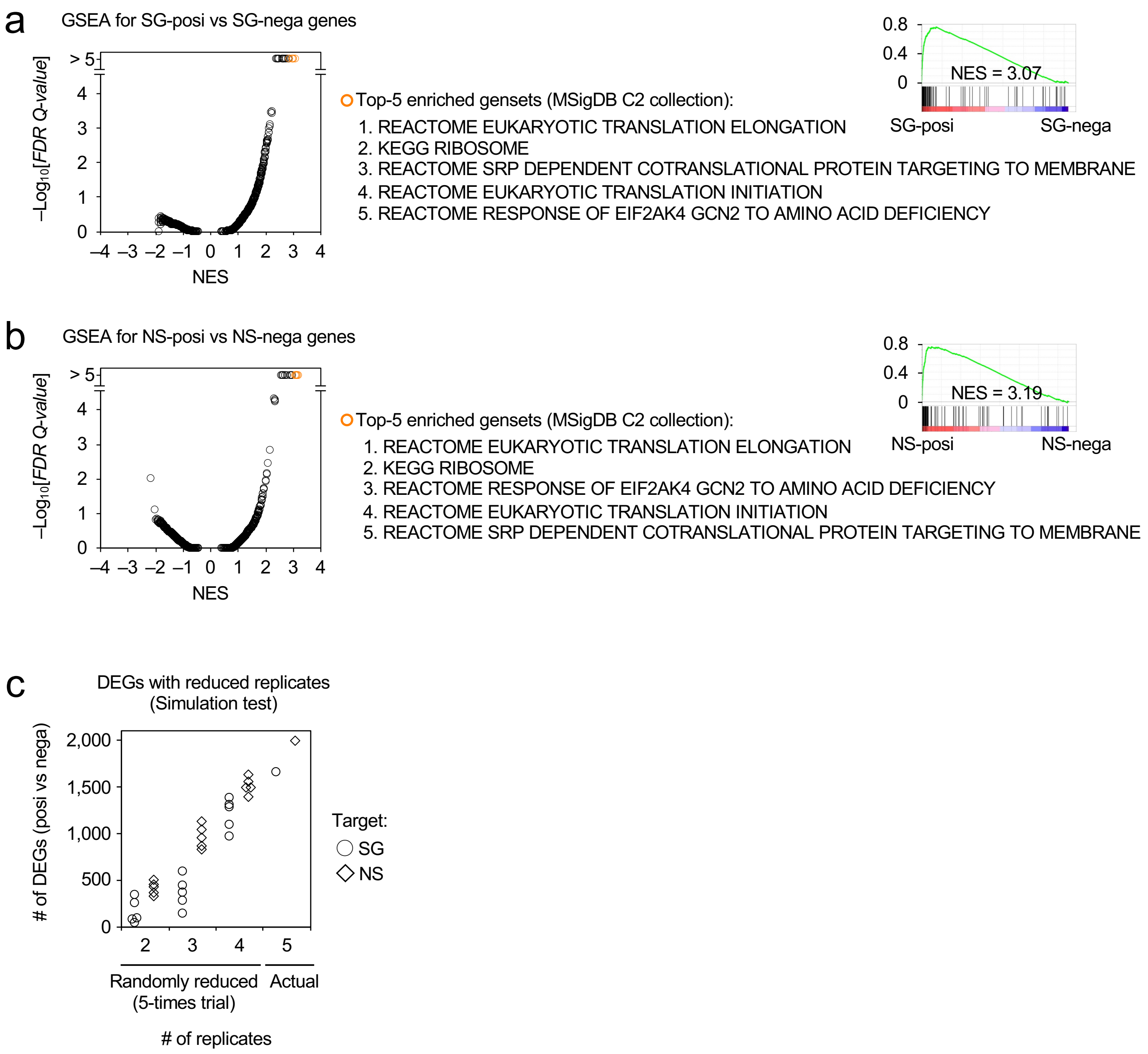

Supplementary Fig. 5 | Features of genes detected from SGs and NSs. a,**b**, GSEA results by the comparison of SG-posi vs SG-nega genes (**a**) and NS-posi vs NS-nega genes (**b**) with the enrichment plots for the most enriched gene set shown on the right. **c**, A simulation test with random reduction of replicates to count the number of DEGs.

**Description**	**Used in:**	**Sequence** (5'→3'; t = NPOM-caged dT; Red = various barcodes; FAM = fluorescein amidite; TMARA = tetramethylrhodamine)
Template for primer extention	Fig. 1b	TCGTCGGCAGCGTCtttGCGTCAGATGTGTATAAGAGACAG
Primer for primer extention	Fig. 1b	TAMRA-CTGTCTCTTATACACATCT
Caged ODN (0 cages)	Fig. 1c	GCCGGTAATACGACTCACTATAGGGTTTGAGTTCTACAGTCCGACGATCNNNNNNAGACTCTTTTTTTTTTTTTTTTTTTTTTTTV
Caged ODN (3 cages)	Fig. 1c	GCCGGTAATACGACTCACTATAGGGTTTGAGTTCTACAGTCCGACGATCNNNNNNCAACTCtttTTTTTTTTTTTTTTTTTTTTTV
Caged ODN (6 cages)	Fig. 1c	GCCGGTAATACGACTCACTATAGGGtttGAGttCtACAGTCCGACGATCNNNNNNAGACTCTTTTTTTTTTTTTTTTTTTTTTTTV
Caged ODN (7 cages)	Fig. 1c	GCCGGTAATACGACTCACTATAGGGtttGAGttCtACAGTCCGACGATCNNNNNNAGCTAGtTTTTTTTTTTTTTTTTTTTTTTV
Caged ODN (8 cages)	Fig. 1c–	GCCGGTAATACGACTCACTATAGGGtttGAGttCtACAGTCCGACGATCNNNNNNTCGAAGttTTTTTTTTTTTTTTTTTTTTTTV
RandomhexRT primer	Fig. 1c, 3, 4, 5, S2	GCCTTGGCACCCGAGAATTCCANNNNNN
RNA PCR primer 1	Fig. 1c, 3, 4, 5, S2	AATGATACGGCGACCACCGAGATCTACACGTTCAGAGTTCTACAGTCCGA
RNA PCR primer 2	Fig. 1c, 3, 4, 5, S2	CAAGCAGAAGACGGCATACGAGATCGTGATGTGACTGGAGTTCCTTGGCACCCGAGAATTCCA
ISH probe for Dcx (forward)	Fig. S2k	GAAATTAATACGACTCACTATAGGTGGAGAGACTGGGCATGGGGA
ISH probe for Dcx (reverse)	Fig. S2k	TGGCAGTGAGCCAAGCCAGA
ISH probe for Hoxb8 (forward)	Fig. S2k	GAAATTAATACGACTCACTATAGGCGCCCTTTTCAGGCGCAGAC
ISH probe for Hoxb8 (reverse)	Fig. S2k	CGCCCCCGCCCCATGTAAAA
ISH probe for Zic1 (forward)	Fig. S2k	GAAATTAATACGACTCACTATAGGTGACACGTAGATCCAGGCTCGT
ISH probe for Zic1 (reverse)	Fig. S2k	CTGAAGGGCGGAAGGGGGTG
ISH probe for Zic4 (forward)	Fig. S2k	GAAATTAATACGACTCACTATAGGTGACACGTAGATCCAGGCTCGT
ISH probe for Zic4 (reverse)	Fig. S2k	CTGAAGGGCGGAAGGGGGTG
Common Forward qPCR primer 1	Fig. 1c	CGACAGGTTCAGAGTTCTACAGTCCGACGATC
Reverse primer for Actb	Fig. 1c	GCTGGCCTGTACACTGACTTGAGACCAATA
Reverse primer for Gapdh	Fig. 1c	ACAATTTCCATCCCAGACCCCCATAATAAC
Reverse primer for Gusb	Fig. 1c	TATGAGCTGGTCCTCCATTTCCCAGGTGAT
Reverse primer for Eef1a	Fig. 1c	AATGGAAACAACTTGACCAAAAATCTGTCAC
Common Forward qPCR primer 2	Fig. 1ef, 2be, S1ac	GCCGGTAATACGACTCACTATAGG
Reverse primer for Gfp	Fig. 1ef, 2b, S1a	GGCATGGACGAGCTGTACAA
TaqMan probe for Gfp	Fig. 1ef, 2b, S1a	FAM-TGGTTACAAATAAAGCAATAGCATCACAAA-TAMRA
Reverse primer for mGapdh	Fig. 2be, S1ac	CAGCAAGGACACTGAGCAAG
TaqMan probe for mGapdh	Fig. 2be, S1ac	FAM-GTGGGTGCAGCGAACTTTATTGA-TAMRA
Reverse primer for hGAPDH	Fig. 2b	CCCCACCACACTGAATCTCC
TaqMan probe for hGAPDH	Fig. 2b	FAM-GTACATGACAAGGTGCGGCTCC-TAMRA
Reverse primer for Sox2	Fig. 2e, S1c	GATTCGGCTCTGTTATTGGAATCAGG
TaqMan probe for Sox2	Fig. 2e, S1c	FAM-TCTCAAACTGTGCATAATGGAGTAAAAACTTAAGT-TAMRA

Supplementary Table | The sequences of oligo DNAs.
