# Supplementary material for: High-depth spatial transcriptome analysis by photo-isolation chemistry
Source: Nat Commun. 2021 Jul 20;12:4416. doi: 10.1038/s41467-021-24691-8 (PMC8292322; doi:10.1038/s41467-021-24691-8)
Supplement: Supplementary file 2 — Reporting Summary [file 41467_2021_24691_MOESM2_ESM.pdf]

nature research

Corresponding author(s): Shinya Oki and Yasuyuki Ohkawa

Last updated by author(s): June 20, 2021

Reporting Summary

Nature Research wishes to improve the reproducibility of the work that we publish. This form provides structure for consistency and transparency in reporting. For further information on Nature Research policies, see our Editorial Policies and the Editorial Policy Checklist.

Statistics

For all statistical analyses, confirm that the following items are present in the figure legend, table legend, main text, or Methods section.

n/a	Confirmed	
☐	☒	The exact sample size (*n*) for each experimental group/condition, given as a discrete number and unit of measurement
☐	☒	A statement on whether measurements were taken from distinct samples or whether the same sample was measured repeatedly
☒	☐	The statistical test(s) used AND whether they are one- or two-sided<br>*Only common tests should be described solely by name; describe more complex techniques in the Methods section.*
☒	☐	A description of all covariates tested
☐	☒	A description of any assumptions or corrections, such as tests of normality and adjustment for multiple comparisons
☐	☒	A full description of the statistical parameters including central tendency (e.g. means) or other basic estimates (e.g. regression coefficient) AND variation (e.g. standard deviation) or associated estimates of uncertainty (e.g. confidence intervals)
☒	☐	For null hypothesis testing, the test statistic (e.g. *F*, *t*, *r*) with confidence intervals, effect sizes, degrees of freedom and *P* value noted<br>*Give P values as exact values whenever suitable.*
☒	☐	For Bayesian analysis, information on the choice of priors and Markov chain Monte Carlo settings
☐	☒	For hierarchical and complex designs, identification of the appropriate level for tests and full reporting of outcomes
☒	☐	Estimates of effect sizes (e.g. Cohen's *d*, Pearson's *r*), indicating how they were calculated

Our web collection on statistics for biologists contains articles on many of the points above.

Software and code

Policy information about availability of computer code

Data collection	Images were taken with Leica DM5000 B and DM6 B fluorescence microscope illuminated with an EL6000 100 W Hg lamp through a Leica HCX objective lens and ImageJ (2.1.0/1.53c) was used to make masking sheets for DMD-assisted UV irradiation.
Data analysis	The sequence reads were aligned to the GRCm38 reference genome with HISAT2 (version 2.1.0) and analyzed using R (3.5.2) packages for read features (featureCount in Rsubread 1.6.3), UMI counting (UMI-tools 0.5.5), dimension reduction (UMAP 0.2.4.1), DEG analysis (DESeq2 1.22.2), and clustering (heatmap3 1.1.6).

For manuscripts utilizing custom algorithms or software that are central to the research but not yet described in published literature, software must be made available to editors and reviewers. We strongly encourage code deposition in a community repository (e.g. GitHub). See the Nature Research guidelines for submitting code & software for further information.

Data

Policy information about availability of data

All manuscripts must include a data availability statement. This statement should provide the following information, where applicable:
- Accession codes, unique identifiers, or web links for publicly available datasets
- A list of figures that have associated raw data
- A description of any restrictions on data availability

The datasets generated during the current study are deposited at GEO under the accession number GSE143413.

Field-specific reporting

Please select the one below that is the best fit for your research. If you are not sure, read the appropriate sections before making your selection.

- [x] Life sciences
- [ ] Behavioural & social sciences
- [ ] Ecological, evolutionary & environmental sciences

For a reference copy of the document with all sections, see nature.com/documents/nr-reporting-summary-flat.pdf

Life sciences study design

All studies must disclose on these points even when the disclosure is negative.

Sample size	Sample size were determined based on similar studies regarding low-input transcritome analysis (Hashimshony, T. et al Genome Biol. 2016; Nichterwitz, S. et al. Nat Commun. 2016) and our experience.
Data exclusions	No data were excluded.
Replication	Each result described in the paper is based on at least three independent biological replicates. All attempts at replication were successful.
Randomization	Groups of cells were randomly allocated into RNA-seq, qPCR, and histological analyses.
Blinding	Investigators were blinded to the group allocation up to the sequencing, qPCR, and histological analyses.

Reporting for specific materials, systems and methods

We require information from authors about some types of materials, experimental systems and methods used in many studies. Here, indicate whether each material, system or method listed is relevant to your study. If you are not sure if a list item applies to your research, read the appropriate section before selecting a response.

Materials & experimental systems

n/a	Involved in the study	
☐	☒	Antibodies
☐	☒	Eukaryotic cell lines
☒	☐	Palaeontology and archaeology
☐	☒	Animals and other organisms
☒	☐	Human research participants
☒	☐	Clinical data
☒	☐	Dual use research of concern

Methods

n/a	Involved in the study	
☒	☐	ChIP-seq
☒	☐	Flow cytometry
☒	☐	MRI-based neuroimaging

Antibodies

Antibodies used	Anti-SOX2 rabbit monoclonal antibody (Cell signaling #23064; 1:1000), anti-G3BP1 antibody (Novus; NBP1-18922; 1:1000), anti-SC35 antibody (Abcam; ab11826; 1:1000), goat anti-rabbit IgG Alexa488 (Invitrogen #A11034; 1:1000), donkey anti-rabbit IgG Alexa555plus (Invitrogen #A32794; 1:1000), goat anti-rabbit IgG Alexa405 (Invitrogen #A48254; 1:250), and donkey anti-mouse IgG Alexa555plus (Invitrogen #A32727; 1:2000).
Validation	Validated by following manufacturer's website (SOX2, https://www.cellsignal.jp/products/primary-antibodies/sox2-d9b8n-rabbit-mab/23064; G3BP1, https://www.novusbio.com/products/g3bp1-antibody_nbp1-18922; SC35, https://www.abcam.com/sc35-antibody-sc-35-nuclear-speckle-marker-ab11826.html).

Eukaryotic cell lines

Policy information about cell lines

Cell line source(s)	Mouse NIH/3T3 and human T-47D and HeLa cells were obtained from ATCC.
Authentication	NIH/3T3 or HeLa and T-47D cells were confirmed to be derived from mouse or human cells, respectively, by qPCR for genes encoding GAPDH.
Mycoplasma contamination	Mycoplasma contamination was not tested.
Commonly misidentified lines (See ICLAC register)	No commonly misidentified cell lines were used.

Animals and other organisms

Policy information about studies involving animals; ARRIVE guidelines recommended for reporting animal research

Laboratory animals	Pregnant wild-type ICR mice (age, not specified) were used to collect embryos at E14.5. 8 week old male mice were used to collect the brains.
Wild animals	No wild animals were used in this study.
Field-collected samples	No field-collected samples were used in this study.
Ethics oversight	The study was approved by the Animal Care and Use Committee of Kyushu University.

Note that full information on the approval of the study protocol must also be provided in the manuscript.
